# Pre-clinical allergenicity assessment of IgE epitope-targeted Der p 2 mutants demonstrate potential as hypoallergenic AIT candidates

**DOI:** 10.3389/fimmu.2025.1623920

**Published:** 2025-06-27

**Authors:** Glorismer Pena-Amelunxen, Mohadeseh Asghari, Kriti Khatri, Jill Glesner, Serge A. Versteeg, Ronald van Ree, Martin D. Chapman, Scott A. Smith, Maksymilian Chruszcz, Anna Pomés, Lorenz Aglas

**Affiliations:** ^1^ Department of Biosciences and Medical Biology, University of Salzburg, Salzburg, Austria; ^2^ Department of Biochemistry and Molecular Biology, Michigan State University, East Lansing, MI, United States; ^3^ Basic Research Department, InBio, Charlottesville, VA, United States; ^4^ Department of Experimental Immunology, Amsterdam University Medical Centers, Amsterdam, Netherlands; ^5^ Department of Otorhinolaryngology, Amsterdam University Medical Centers, Amsterdam, Netherlands; ^6^ Department of Medicine, Vanderbilt University Medical Center, Nashville, TN, United States; ^7^ Institute of Pathophysiology and Allergy Research, Center for Pathophysiology, Infectiology and Immunology, Medical University of Vienna, Vienna, Austria; ^8^ Human Microbiome (HUMI) Research Program, Faculty of Medicine, University of Helsinki, Helsinki, Finland

**Keywords:** allergen-specific immunotherapy, house dust mite, Der p 2, hypoallergenic, human IgE monoclonal antibodies

## Abstract

**Background:**

Advancements in hybridoma technology have enabled the production of human IgE monoclonal antibodies (hIgE mAb) for successful IgE epitope mapping of major allergens. Here, we assessed the hypoallergenicity of three IgE-epitope mutants (single 4C8 or 2F10, and double 4C8 + 2F10 epitope mutants) of house dust mite allergen (HDM) Der p 2.

**Methods:**

Humanized rat basophilic leukemia (huRBL) cells, passively sensitized overnight with either pairs of Der p 2 specific hIgE mAb (2F10, 4C8 or 2G1) or HDM-allergic serum (n=8), were stimulated with either wildtype (WT) Der p 2 or an epitope mutant and mediator release was measured.

**Results:**

No degranulation was induced upon stimulation with all mutants, when cells were sensitized with pairs of hIgE mAb specific for at least one mutated epitope. HIgE mAb specific for non-mutated epitopes led to mediator release comparable to WT Der p 2, indicating that epitopes recognized by the three different hIgE mAb are not overlapping and that the 3D-structure of the mutants is conserved. The double 4C8 + 2F10 epitope mutant had a significantly reduced maximal mediator release (48.3%) compared to the WT, in cells sensitized with allergic donor serum. Overall, the area-under-the-curve of mediator release curves induced by the mutants was significantly lower (31-65%) compared to WT. When comparing the EC_20_, the double 4C8 + 2F10 epitope mutant required a 158-fold higher antigen concentration to induce the same extent of mediator release as WT Der p 2.

**Conclusion:**

Der p 2 epitope mutants display significantly reduced allergenicity. Particularly, the double 4C8 + 2F10 epitope mutant demonstrated a strong potential as a novel AIT vaccine candidate.

## Introduction

House dust mites (HDM) are among the most prevalent sources of indoor allergens globally, with an estimated 1–2% of the world population sensitized to HDM allergens (1)*. Dermatophagoides pteronyssinus* (Der p) and *Dermatophagoides farinae* (Der f) are the primary species implicated in HDM related allergic conditions. Allergenic proteins from groups 1 (e.g., Der p 1) and 2 (e.g., Der p 2) are recognized as major allergens in HDM sensitization. Approximately 90% of individuals sensitized to *Dermatophagoides* allergens show specific IgE to Der p 1 and/or Der p 2, underlining their central role in allergic reaction*s* (2). A 2016 longitudinal study further highlighted the clinical importance of Der p 2, demonstrating that IgE sensitization to this allergen during childhood strongly predicts the development of asthma later in life (3). Given the widespread impact of these allergens, there is ongoing research aimed at developing more effective therapeutic strategies for HDM-allergic patients.

Recent advancements in hybridoma technology, involving the immortalization of IgE-producing B cells, have enabled detailed IgE epitope mapping on allergens (4–9). A method introduced by Wurth et al. results in the efficient generation of hIgE mAb from the blood of allergic individuals (10). This process preserves the natural heavy and light chain pairing of antibodies, facilitating the study of IgE-binding epitopes in a clinically relevant context. Among the hIgE mAb developed are the three Der p 2-specific hIgE mAb 2F10, 2G1 and 1B8 (4), of which, the combination 2F10 and 2G1 was found to be biologically functional in inducing effector cell mediator release and anaphylaxis in mice *in vivo*, upon allergen exposure (4, 7, 8, 11). The hIgE mAb 2F10 was found to bind specifically to an epitope that was recognized by IgE from 10 tested plasmas from allergic subjects (7).

Allergen-specific hIgE mAb not only facilitate advancements in allergy diagnostics but also pave the way for innovative approaches in allergy immunotherapy (AIT), currently the only treatment for IgE-mediated allergies achieving a long-term relief of symptoms. AIT works by administrating (subcutaneously or sublingually) high doses of allergens in a period of 3–5 years to induce immunotolerance in the patients. Limitations of AIT include poor compliance, in part due to the occurrence of side effects which can be as severe as inducing anaphylaxis in AIT recipients (12). A strategy to circumvent such side effects is the development of hypoallergens. A key element of hypoallergen design is to specifically modify allergens to keep their immunogenic properties (i.e. ability to trigger an adaptive immune response), while reducing their allergenicity by destruction of IgE epitopes, therefore, reducing IgE crosslinking and the release of symptoms-inducing mediators (9, 13).

The previously mentioned hIgE mAb 2F10 has been applied for the development of Der p 2 hypoallergens. The analysis of the X-ray crystal structure of Der p 2 complexed with the Fab region of the hIgE mAb 2F10 enabled the identification of amino acid residues comprising the hIgE mAb 2F10 binding epitope (7). By introducing specific amino acid substitutions on the epitope, a hypoallergenic variant of wildtype Dep 2 was produced, that was not recognized by the hIgE mAb 2F10 and displayed a significantly reduced reactivity with polyclonal plasma from HDM-allergic individuals (7). By employing the same strategy using an hIgE mAb targeting a distinct epitope on Der p 2, 4C8 (8), another Der p 2 epitope mutant was created. In parallel, mutations in both 2F10 and 4C8 epitopes generated a double epitope mutant. Each of the three mutants demonstrated a complete abrogation of binding to their respective hIgE mAb and a markedly diminished IgE binding from mite-allergic individuals’ plasma (7, 8).

**Figure 1:**
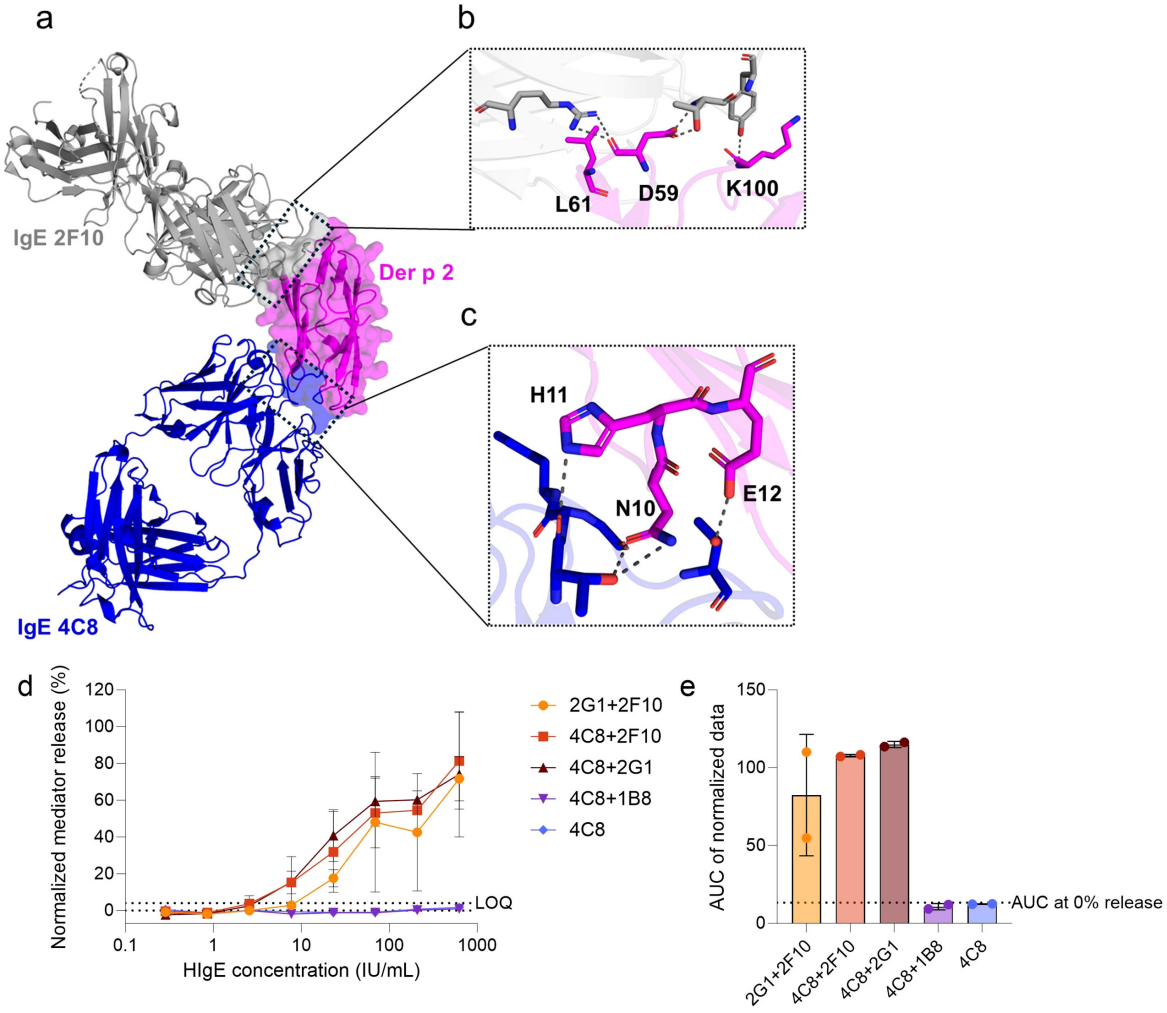
Screening of functional capacity of hIgE mAb combinations to sensitize huRBL cells and induce degranulation when stimulated with WT Der p 2. **(a)** Superimposed crystal structures of Der p 2 in complex with hIgE mAb 2F10 (PDB: 7MLH) and Der p 2 in complex with hIgE mAb 4C8 (PDB: 8VK1). HIgE mAb 2F10 (in grey) and hIgE mAb 4C8 (in blue) epitopes on Der p 2 (in magenta) surface are marked by the black dotted boxes. The enlarged boxes display the Der p 2 epitope residues targeted to design **(b)** the single 2F10 epitope mutant (D59K-L61K, and K100D) and **(c)** the single 4C8 epitope mutant (N10A-H11A-E12S). Cells were sensitized with a dilution series of hIgE mAb starting at a concentration of 625 IU/mL, followed by 1:3 dilutions, and stimulated with 1 µg/mL of WT Der p 2. Normalized mediator release curves are expressed in percentages **(d)** and AUC values were calculated thereof **(e)**. The hIgE mAb combinations used for cell´s sensitization is indicated in the graph´s legend and axis label. LOQ for the mediator release curve graph and AUC at 0% mediator release are expressed as dotted lines as reference values. Error bars represent the standard deviation. * (p<0.05), ** (p<0.01), *** (p<0.001).

This study aimed to investigate the allergenicity of the Der p 2 single 4C8 or 2F10 epitope mutants, as well as the double 4C8 + 2F10 epitope mutant (**Figures 1a–c**), in comparison to the wildtype Der p 2 reference. Recombinant Der p 2.0103 was used as wildtype allergen reference, termed WT Der p 2, since this isoform served as template for site-directed mutagenesis to generate the recombinant point mutants (7, 8). Specifically, the functionality of the Der p 2 mutants in cross-linking IgE (hIgE mAb or polyclonal IgE from HDM allergic donor serum) bound to humanized rat basophilic leukemia (huRBL) cells and inducing mediator release was assessed. The objective was to evaluate if disruption of the two disease-relevant IgE epitopes by targeted mutations results in lowered allergenicity compared to the WT allergen.

## Methods

### Production of Der p 2 IgE-epitope mutants

The rational design for site-directed mutagenesis of the epitopes aimed at disrupting hydrogen bonds or hydrophobic interactions, by changing the size or charge of the amino acid residues involved in interactions, as previously described (7, 8). The Der p 2 epitope mutants were expressed in *Pichia pastoris* and purified as reported (7, 8). Briefly, DNA encoding recombinant Der p 2.0103 was synthesized to include the following mutations: N10A, H11A, and E12S for the single 4C8 epitope mutant, D59K, L61K, and K100D for the single 2F10 epitope mutant, and N10A, H11A, E12S, D59K, and L61K for the double 4C8 + 2F10 epitope mutant. The DNA encoding the mutants was cloned into the pPICZαA vector with a N-terminal 6xHis-tag by Genscript (Piscataway, NJ, USA). The plasmid was linearized and transformed into *Pichia pastoris* for recombinant gene expression of the mutants using methanol induction. The Der p 2 mutants were purified using a HisTrap™ HP 5 mL column (Cytiva, Marlborough, Massachusetts, USA). Proof of correct folding of the mutants was previously reported (7, 8).

### Passive sensitization of huRBL cells

To compare the allergenicity of the mutants to the WT Der p 2, the huRBL cell mediator release assay was deployed and performed as previously described (11). Briefly, overnight passive IgE sensitization of huRBL cells with either pairs of hIgE mAb or serum derived from European HDM-allergic patients. Selection of hIgE mAb and passive sensitization protocol can be found in the **Supplementary Methods**, mAb and serum information can be found in **Supplementary Tables 1, 2**. The following day, cells were stimulated with allergen for 1 h at 37°C to induce cross-linking of the IgE on the huRBL cells. After allergen stimulation, the supernatants were collected and added to an assay buffer containing the substrate 4-methylumbelliferyl β-D-glucuronide dihydrate (4MUG, Sigma, Darmstadt, Germany), for the detection of the mediator release proxy, β-hexosaminidase.

### Allergen stimulation of sensitized huRBL cells

The stimulation of IgE-sensitized huRBL cells with recombinant WT Der p 2 and mutants (that will be referred to as single 4C8 or single 2F10 epitope mutants and double 4C8 + 2F10 epitope mutant) was compared (listed in **Supplementary Table 3**). Allergen concentrations ranged from 1 to 1 × 10^-7^ µg/mL. To assess the contribution to allergenicity of another epitope non-overlapping with the epitopes for IgE mAb 2F10 and 4C8, WT Der p 2 and mutants thereof were pre-incubated with the Der p 2-specific murine IgG monoclonal antibody (mIgG) α-DpX (14, 15). This antibody epitope is known to overlap with epitopes of hIgE mAb 5D10, 1B8 and 2G1 (4). The mIgG was incubated with WT Der p 2 and mutants for 1 h at 37°C prior to cell stimulation to facilitate antibody-antigen binding. The mIgG α-DpX was used at a 1:10 serial dilution starting at 20 µg/mL, or at a constant concentration of 2 µg/mL, depending on the experimental conditions. HuRBL mediator release assay controls and data analysis and statistics can be found in the **Supplementary Methods**.

## Results

### Der p 2 IgE-epitope mutants exhibit reduced potency to trigger hIgE mAb-mediated degranulation

First, hIgE mAb pairs were evaluated for their ability to sensitize huRBL cells efficiently and to induce degranulation upon stimulation with WT Der p 2. Using the huRBL mediator release assay, four hIgE mAb pairings were screened to assess their cross-linking potential: 2G1 + 2F10, 4C8 + 2F10, 4C8 + 2G1, and 4C8 + 1B8 (**Figures 1d, e**). Sensitization with hIgE mAb 4C8 alone served as a negative control. Of note, in our previous study, the individual sensitization with hIgE mAb 2F10, 2G1, and 1B8, as well as pairings such as hIgE mAb 2G1 + 2F10, 2G1 + 1B8, and 2F10 + 1B8 were investigated (11). Among these, the hIgE mAb 2G1 + 2F10 combination elicited the highest mediator release and was included in this study for comparison. Screening revealed that three hIgE mAb pairs — 2G1 + 2F10, 4C8 + 2F10, and 4C8 + 2G1 — induced comparable and significant mediator release (>60% maximum release) upon stimulation with WT Der p 2 (**Figure 1d**). In contrast, the hIgE mAb 4C8 control and 4C8 + 1B8 pairing showed no relevant mediator release. AUC analysis of mediator release curves revealed that hIgE mAb 4C8 + 2F10 (AUC: 107.8) and 4C8 + 2G1 (AUC: 115.0) slightly outperformed 2G1 + 2F10 (AUC: 82.4) by 23.5% and 28.3%, respectively (**Figure 1e**).

**Figure 2:**
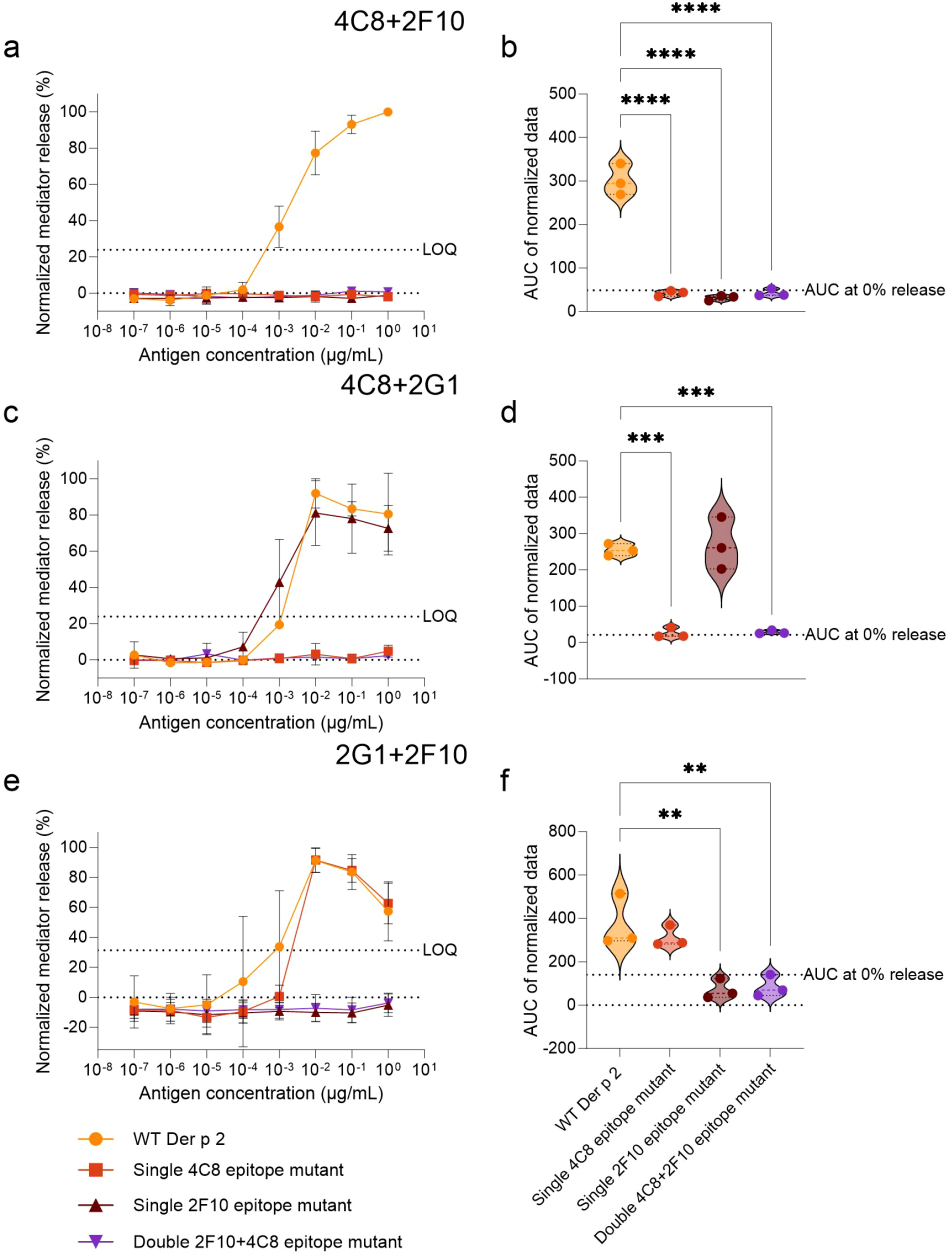
Comparison of Der p 2 IgE-epitope mutants with the WT in stimulating mediator release from huRBL cells sensitized with functional hIgE mAb combinations. Cells were sensitized with hIgE mAb at a concentration of 70 IU/mL and stimulated the following day with a starting concentration of 1 µg/mL of either WT or Der p 2 single and double epitope mutants followed by a 1:10 titration. The combinations of hIgE mAb used for sensitization are indicated on top of the respective graphs. Normalized mediator release curves are expressed in percentages **(a**, **c**, **e)** and an AUC analysis was performed **(b, d, f)**. LOQ for the mediator release curve graph and AUC at 0% mediator release are represented by dotted lines as reference values. Ordinary one-way ANOVA analyses with multiple comparisons were done to determine statistically significant differences between the AUC of the WT and each IgE-epitope mutant **(b**, **d**, **f)**. ** (p<0.01), *** (p<0.001), **** (p<0.0001).

To validate the effects of the specific epitope mutations in disrupting IgE binding, WT Der p 2 and mutants thereof were screened for their capacity to induce mediator release in hRBL cells sensitized with hIgE mAb pairs (**Figure 2**). All mutants lacked the ability to induce degranulation in huRBL cells sensitized with hIgE mAb corresponding to their mutated epitopes. However, certain sensitizing hIgE mAb combinations led to mediator release comparable to WT Der p 2, such as IgE mAb 2G1 + 4C8 (WT AUC: 254.8 *vs*. single 2F10 epitope mutant AUC: 269.7) and hIgE mAb 2G1 + 2F10 (WT AUC: 373.9 *vs*. single 4C8 epitope mutant AUC: 313.3), indicating that the three epitopes to which the individual hIgE mAb 2G1, 4C8 and 2F10 bind to are not overlapping and their IgE binding capabilities remain intact (**Figures 2b, d, f**). Statistical analyses confirmed significantly reduced AUCs below the threshold for mediator release when cells were sensitized with mutant-specific hIgE mAb pairs (p < 0.001, p < 0.0001, p < 0.00001).

### Reduced allergenicity of Der p 2 IgE-epitope mutants using HDM-allergic donor sera

**Figure 3:**
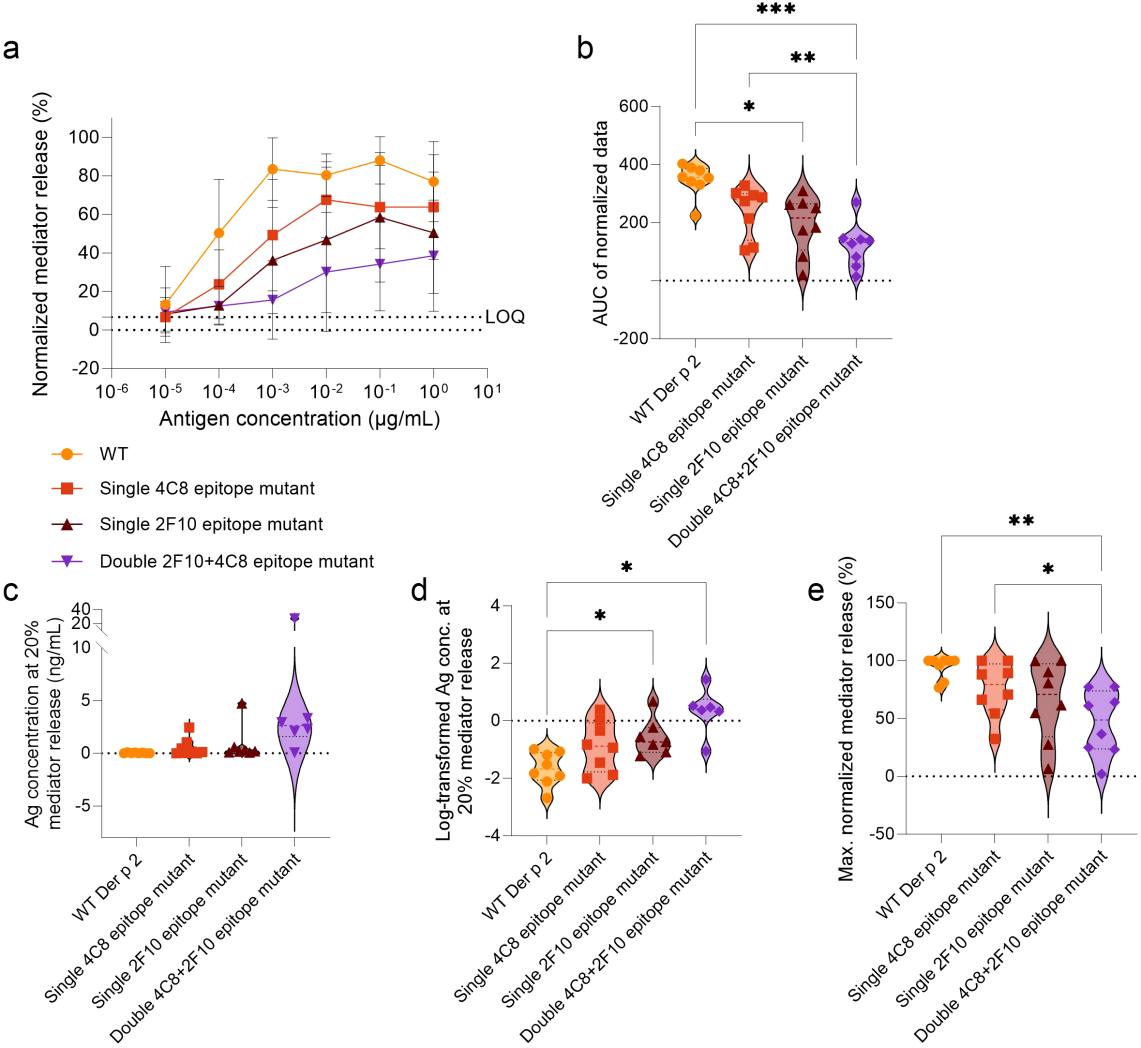
Comparison of Der p 2 IgE-epitope mutants versus WT in stimulating mediator release from huRBL cells sensitized with serum from HDM allergic donors. Cells were sensitized with serum from HDM allergic donors (n = 8) diluted 1:20 in huRBL cell medium and stimulated the following day with a starting concentration of 1 µg/mL of antigen followed by a 1:10 titration. Normalized mediator release curves are expressed in percentages **(a)** and an AUC analysis was performed **(b)**. LOQ for the mediator release curve graph is represented by dotted line as reference. The antigen and log_10_-transformed antigen concentration required to obtain 20% mediator release [EC_20_, **(c, d)**, respectively]. Lastly, maximum normalized mediator release percentages were compared **(e)**. Repeated measures one-way ANOVA analyses with multiple comparisons were done to determine statistically significant differences. * (p<0.05), ** (p<0.01), *** (p<0.001), **** (p<0.0001).

Using sera from HDM-allergic donors, the potency of each mutant to induce mediator release in comparison to WT Der p 2 was evaluated in a clinically relevant context (**Figure 3**). All Der p 2 IgE-epitope mutants demonstrated reduced mediator release compared to WT Der p 2 (**Figures 3a–d**). AUC analysis supported these findings, with WT (AUC: 347.7) significantly outperforming the single 2F10 epitope mutant (AUC: 193.2) and the double 4C8 + 2F10 epitope mutant (AUC: 121.4), p < 0.01 and p < 0.0001, respectively (**Figure 3b**). There was no significant difference between the AUC of the single 4C8 epitope mutant and that of the WT. The average AUC in all mutants was reduced by 31% (single 4C8 epitope mutant), 44.4% (single 2F10 epitope mutant) and 65.1% (double 4C8 + 2F10 epitope mutant), when compared to the WT. Additionally, double 4C8 + 2F10 epitope mutant had a significantly lower AUC compared to the single 4C8 epitope mutant (AUC: 239.8; p < 0.01). To further compare differences in allergen potency, the antigen concentrations required for 20% mediator release (EC_20_) were analyzed (**Figures 3c–d**). WT required the lowest concentration (0.04 ng/mL), followed by the single 4C8 epitope mutant (0.53 ng/mL), the single 2F10 epitope mutant (0.86 ng/mL), and the double 2F10 + 4C8 epitope mutant (6.36 ng/mL), indicating that a 12-, 21- and 158-fold higher antigen concentration, respectively, is required to induce the same degree of mediator release. Log_10_-transformation of these values highlighted significant differences between WT and the single 2F10 epitope mutant and the double 4C8 + 2F10 epitope mutant (p < 0.05, for both, **Figures 3d**).

The WT allergen elicited the highest average maximal mediator release (94.3%), followed by the single 4C8 epitope mutant (75.2%), the single 2F10 epitope mutant (65%), and the double 4C8 + 2F10 epitope mutant (45.9%) (**Figure 3e**). Notably, the double 4C8 + 2F10 epitope mutant had a higher mean reduction in percentage maximal mediator release (ΔWT-mutant), by 48.3%, compared to WT (p < 0.01) and 29.3%, compared to the single 4C8 epitope mutant (p < 0.05).

Additionally, donor serum 5 induced mediator release exceeding the LOQ threshold when stimulated with all the antigens. However, all the mutants produced maximum release below 35%, while the WT Der p 2 induced a maximum of 100% mediator release (**Supplementary Figure 1**; **Supplementary Table 4**). Donor serum 6 produced low maximum release of 24.9% when stimulated with the double 4C8 + 2F10 epitope mutant, while the single epitope mutants induced mediator release comparable to the WT. Donor serum 7 did not induce any mediator release exceeding the LOQ threshold when stimulated with either the single 2F10 epitope mutant or the double 4C8 + 2F10 epitope mutant. Donor serum 4 could also be considered a non-responder when stimulated with the double 4C8 + 2F10 epitope mutant since the mediator release curve is above the LOQ due to a baseline-drift, resulting in a bottom plateau (of all samples) which is higher than the LOQ. Altogether, it became evident that for 4 of 8 patients (50%) there was no induction of any relevant mediator release when cells were stimulated with the double 4C8 + 2F10 epitope mutant, which indicates a complete loss of allergenicity in these patients (**Supplementary Figure 1**).

These data are in line with our previously reported inhibition ELISA data (7) showing that a 38-fold higher antigen concentration was required of the single 2F10 epitope mutant to achieve the same level of inhibition of the polyclonal IgE response of mite-allergic patients as WT Der p 2 (0.2050 µg/mL *vs*. 0.0054 µg/mL, **Supplementary Figure 2**).

### Murine IgG mAb αDpX showed the capacity to attenuate IgE cross-linking by WT Der p 2 and the single 2F10 epitope mutant but not by the other mutants in huRBL cells sensitized with an HDM-allergic serum pool

**Figure 4:**
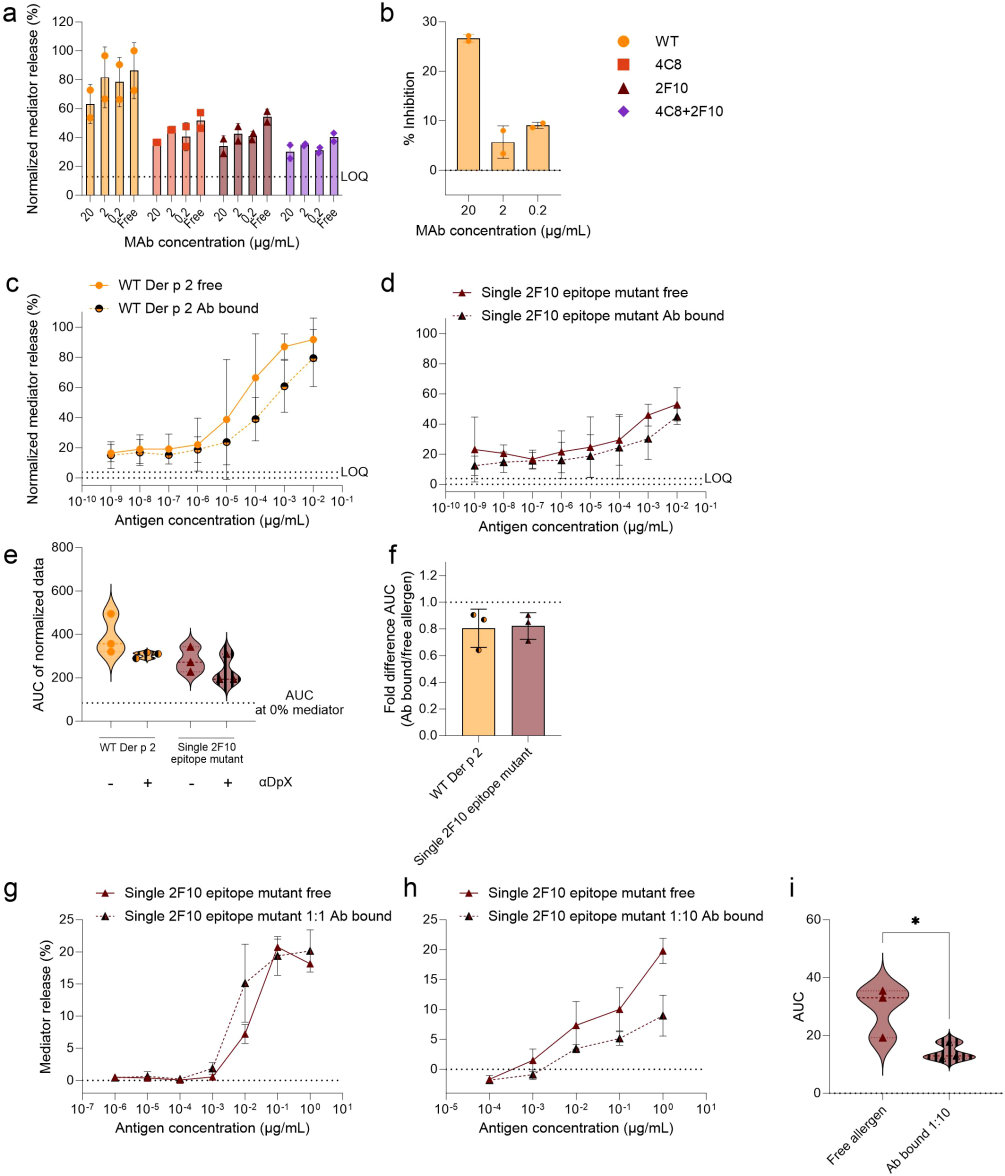
Analysis of potential of mIgG mAb α-DpX to bind to a non-mutated epitope on WT Der p 2 and the IgE-epitope mutants. HuRBL cells were sensitized using a serum pool of previously tested HDM allergic donor serum, diluted 1:20 in huRBL cell medium. The following day, all antigens, either free allergen or bound by mIgG mAb α-DpX, were used to stimulate huRBL cells and to induced mediator release. MIgG mAb α-DpX was titrated to determine the optimal α-DpX-antigen molar ratio for mediator release inhibition (**a**, **b**). Using mIgG mAb α-DpX at a constant concentration of 2 µg/mL to bind different concentrations of Der p 2 antigens, 1:10 dilution series, starting at 100 ng/mL, representing a 1:1 molar ratio of α-DpX-to-antigen. Normalized mediator release was expressed as percentages **(c, d)** and an AUC analysis was performed **(e)**. The fold difference of α-DpX-bound and free allergen was calculated **(f)**. Cells sensitized with hIgE mAb 4C8 + 2G1 were stimulated with single 2F10 epitope mutant bound to α-DpX with a 1:1 **(g)** and a 1:10 **(h)** molar ratio (antigen-to-α-DpX). AUC analysis was performed to compare the curves from the free allergen and the allergen bound to α-DpX in a 1:10 molar ratio **(i)**, unpaired t test was used to determine statistically significant differences. LOQ for the mediator release curve graph and AUC at 0% mediator release are represented by dotted lines as reference values. * (p<0.05).

To assess the influence of other non-mutated IgE epitopes on the allergenicity of Der p 2, the mIgG mAb α-DpX was used, as it has been reported to not recognize any overlapping amino acid residues with the 2F10 epitope but has at least some overlap with the 2G1 epitope. An overlap of epitopes for murine IgG mAb α-DpX and hIgE mAb 2G1 was previously reported by nuclear magnetic resonance and via two-site and inhibition ELISAs (4). Therefore, inhibition assays were performed using the mIgG mAb α-DpX bound to either WT Der p 2 or the mutants. The rationale of using IgG mAb α-DpX inhibition as surrogate for a single 2G1 epitope mutant, which was not available (since the structure of Der p 2 in complex with hIgE mAb 2G1 had not been determined yet), was to indirectly assess the relevance of the 2G1 epitope in IgE binding and induction of mediator release in huRBL cells. WT Der p 2 and mutants were pre-incubated with mIgG mAb α-DpX before being used to stimulate mediator release in huRBL cells passively sensitized with a serum pool. To optimize the experimental setup, mIgG mAb α-DpX was titrated in a 1:10 series from 20 µg/mL to 0.2 µg/mL, with antigens added at a final concentration of 1 µg/mL (**Figure 4a**). Here, the highest concentration of α-DpX, representing a 1:1 molar ratio of mIgG mAb to antigen (20 µg/mL mAb and 1 µg/mL antigen), was identified as the most effective, achieving an average inhibition exceeding 25% for the WT Der p 2 (**Figure 4b**). Subsequently, cells were stimulated with titrated concentrations of the free antigens (WT or mutants). For these experiments, mIgG mAb α-DpX-antigen concentrations were titrated in a 1:10 series starting at 100 ng/mL (representing a 1:1 molar ratio of mIgG mAb to antigen concentration). Differences were observed between the mediator release produced when stimulating with the free and antibody-bound WT Der p 2 and the single 2F10 epitope mutant (**Figures 4c, d**), but not the single 4C8 epitope mutant and the double 4C8 + 2F10 epitope mutant (**Supplementary Figure 3**). When determining the fold difference between the average AUC produced by the free and antibody bound allergen, WT Der p 2 and single 2F10 epitope mutant showed a 19.5% and 17.8% reduction in AUC, respectively, when bound to α-DpX (**Figures 4e, f**). This reduction in allergenicity of the antibody-bound single 2F10 epitope mutant let us to further investigate this inhibition in huRBL cells sensitized with the hIgE mAb combination 4C8 + 2F10(**Figures 4g–i**). When the single 2F10 epitope mutant was bound to the αDpX at a 1:1 molar ratio, only a slight decrease in the mediator release curve was observed (**Figure 4g**). After increasing the allergen-to-αDpX molar ratio to 1:10, we saw a flattening of the mediator release curve (**Figure 4h**). A comparison of the AUC showed a significant reduction in AUC of 51.2% with αDpX bound to the single 2F10 epitope mutant when compared to the free mutant (p < 0.05) (**Figure 4i**). Taken together these data indicate that αDpX-binding reduced the degranulation potential of WT Der p 2 and the single 2F10 epitope mutant but not of the other two mutants.

## Discussion

Hypoallergens have the potential to make AIT safer and more effective. This study investigated the hypoallergenicity of three Der p 2 mutants within a preclinical safety evaluation. The results demonstrate a complete elimination in the capacity to induce mediator release in cells passively sensitized with hIgE mAb that have epitope specificities corresponding to the mutated epitopes. Additionally, the unaffected recognition of unmutated epitopes by their respective hIgE mAb provided evidence of proper folding of the mutants, especially since the epitopes recognized by the IgE mAb are known to be conformational (7, 8). Proper protein folding of the mutants is important for allergen recognition of the immune system and the production of specific “blocking” IgG antibodies, able to interfere with IgE-allergen binding (16).

When cells were sensitized with serum from allergic donors, the Der p 2 IgE-epitope mutants had reduced capacity to stimulate huRBL cell degranulation versus the WT. Interestingly, the single 4C8 epitope mutant was the only mutant whose allergenicity, tested using the allergic donor serum as sensitizer, was not significantly reduced, although a trend in mediator release reduction was observed. This result indicates that the 4C8 epitope is a less relevant IgE epitope compared to the 2F10 epitope within the tested patients’ specific IgE repertoire. The double 4C8 + 2F10 epitope mutant induced a 35% reduced mediator release and a 51.4% reduction in maximum mediator release versus the WT when comparing the AUC of the release curves. Moreover, the double 4C8 + 2F10 epitope mutant required a 158-fold higher concentration to induce the EC_20_. These results agree with those previously reported from binding assays and *in vivo* passive anaphylaxis transgenic mouse model (7, 8). Like the *in vivo* passive anaphylaxis transgenic mouse model, the mediator release assay allowed us to test the capacity for hIgE mAb-crosslinking and effector cell degranulation, with the advantage of having performed titrations and showing dose-dependent response curves in the current study. This dose-dependent response provides insights into the degree of reduction in allergenicity achieved with the mutants, which is highly important when assessing the pre-clinical safety of a potential AIT candidate. For example, by screening the mutants with the serum from allergic donors, it is possible to determine which donor would benefit from treatment with these hypoallergens. From this study, it can be speculated that donors 5, 6 and 7 would experience a low allergenic risk when undergoing AIT using the double 4C8 + 2F10 epitope mutant, enabling a quicker up-dosing since the risk of occurrence of adverse events is expected to be reduced.

Previous publications have reported a variety of strategies to produce HDM hypoallergens. One of them involved creating hybrid proteins made by a combination of fragments from Der p 2 and other major and minor allergens (17, 18). Similarly, another study reported a hybrid protein containing previously published T-cell epitopes of the Der p 1, Der p 2 and Der p 23 allergens, aiming to push the immune response to these proteins towards a more balanced Th1/Th2 response (19). Unlike those hybrid proteins, the advantage of the mutants in the current study is that they present the naturally occurring folded protein structure, which allows for the immune system to develop tolerance to the “natural” epitopes. However, the influence of site-directed mutagenesis on T cell immunogenicity of the single and double IgE-epitope mutants in comparison to WT Der p 2 must be addressed in-depth in future studies.

Another study reported a Der p 2 hypoallergen produced by engineering a point mutation at residue 47 (S47W), which prevented IgE binding, but also changed the protein secondary structure (20). The mutants tested in the current study were designed based on the crystal structures of Der p 2 in complex with hIgE mAb, providing the advantage of knowing the exact residues involved in IgE-Der p 2 binding. This allowed a rational design of targeted site-directed mutagenesis, while preserving structurally important residues (7, 8). This approach facilitates the development of hypoallergens that retain the overall structural integrity of the protein.

Immunodominant epitopes have been reported in various allergens. In the major peanut allergen Ara h 2 the immunodominant epitope containing DPYSP^OH^S motifs account for approximately 90% of IgE binding in polyclonal human serum (21). We observed a 44.4% reduction in allergenicity of the single 2F10 epitope mutant versus WT Der p 2, assessed as mediator release from hRBL cells passively sensitized with serum from HDM allergic donors. When comparing the allergenicity of the single 4C8 and single 2F10 epitope mutants, the single 2F10 epitope mutant is the only one of the two to have a significant reduction in allergenicity from the WT. Based on our data, we assume that among the two IgE epitopes, 2F10 exhibits greater immunodominance. Nevertheless, as the double 4C8 + 2F10 epitope mutant demonstrates the most pronounced reduction in allergenicity, it is obvious that both epitopes contribute significantly to the IgE reactivity.

Our attempt to further reduce the allergenicity of the mutants by using the mIgG mAb α-DpX, known to overlap with the hIgE mAb 2G1 epitope (4), did not result in further reduction of mediator release induced by the single 4C8 epitope mutant or the double 4C8 + 2F10 epitope mutant. We can speculate that the mIgG mAb α-DpX epitope either has some overlap with the 4C8 epitope or the proximity of both epitopes prevent cross-linking (15). This may explain the lack of inhibition by α-DpX in Der p 2 IgE-epitope mutants that have a mutated 4C8 epitope. Another possible explanation for the lack of mediator release inhibition for the double 4C8 + 2F10 epitope mutant is that the allergenicity of the double epitope mutant cannot be further reduced. The α-DpX-inhibited WT Der p 2 had almost a 20% reduction in allergenicity when compared to the free WT, similarly to the reduced allergenicity observed for the single 4C8 and single 2F10 epitope mutants. Additionally, the allergenicity of the single 2F10 epitope mutant was significantly reduced after inhibition with α-DpX. Instead of using the mIgG mAb α-DpX for inhibition, it might be worthwhile investigating in the future whether a single 2G1 epitope mutant or a triple 4C8 + 2F10 + 2G1 epitope mutant could provide other hypoallergenic AIT candidates.

This study demonstrated the reduced allergenicity of Der p 2 hypoallergens, compared to the WT allergen. This investigation is intended to extend earlier studies that developed IgE-epitope mutants based on the crystal structures of allergen-IgE antibody complexes (7, 8). Here, an analysis of the allergenicity of WT Der p 2 and thereof derived hypoallergens was performed using mediator release assays across a broader allergen concentration range. The results highlight the potential suitability of the hypoallergens – especially the double 4C8 + 2F10 epitope mutant – for AIT due to their reduced IgE-reactivity in human serum from HDM allergic donors. We also found that the epitope recognized by the mIgG mAb αDpX may be a suitable target for mutation to produce another hypoallergenic candidate. Additionally, we highlight the usage of single epitope mutants in profiling the IgE repertoire of polyclonal patient´s sera. Overall, using hIgE mAb to elucidate which amino acid residues on allergens are responsible to IgE-antigen binding facilitates a more targeted (or even patient-driven) approach to producing hypoallergens for AIT treatments. Confirmation for such approach is provided by the highly reduced IgE reactivity of the double 4C8 + 2F10 epitope mutant in our study. These findings bring us a step forward in testing these hypoallergenic Der p 2 mutants in a first-in-human clinical trial as a novel AIT vaccine candidate.

## Data Availability

The raw data supporting the conclusions of this article will be made available by the authors, without undue reservation.
