# Supplementary material for: Pre-clinical allergenicity assessment of IgE epitope-targeted Der p 2 mutants demonstrate potential as hypoallergenic AIT candidates
Source: Front Immunol. 2025 Jun 27;16:1623920. doi: 10.3389/fimmu.2025.1623920 (PMC12246809; doi:10.3389/fimmu.2025.1623920)
Supplement: Supplementary file 1 [file DataSheet1.pdf]

Pre-clinical allergenicity assessment of IgE epitope-targeted Der p 2 mutants demonstrate potential as hypoallergenic AIT candidates

Glorismer Pena-Amelunxen[1], Mohadeseh Asghari[1], Kriti Khatri[2], Jill Glesner[3], Serge A. Versteeg[4,5], Ronald van Ree[4,5], Martin D. Chapman[3], Scott A. Smith[6], Maksymilian Chruszcz[2], Anna Pomés[3*], Lorenz Aglas[1,7,8*]

[1]Department of Biosciences and Medical Biology, University of Salzburg, Salzburg Austria
[2]Michigan State University, East Lansing, MI, United States
[3]InBio, Charlottesville, VA, United States
[4]Department of Experimental Immunology, Amsterdam University Medical Centers, Netherlands
[5]Department of Otorhinolaryngology, Amsterdam University Medical Centers, Amsterdam, Netherlands
[6]Department of Medicine, Vanderbilt University Medical Center, Nashville, TN, United States
[7]Institute of Pathophysiology and Allergy Research, Center for Pathophysiology, Infectiology and Immunology, Medical University of Vienna, Vienna, Austria
[8]Human Microbiome (HUMI) Research Program, Faculty of Medicine, University of Helsinki, Helsinki, Finland

***Correspondence:**
Lorenz Aglas
ORCID: 0000-0002-1236-5934
E-mail: lorenz.aglas@helsinki.fi

Anna Pomés
ORCID: 0000-0002-8729-1829
E-mail: apomes@inbio.com

Supplementary Methods

Selection of hIgE mAb

hIgE mAb specific for Der p 2 were produced and purified as previously described[1–4]. In short, hIgE mAb were produced by cytofusion of B cells from HDM allergic donors, who donated at the allergy clinic of the Vanderbilt University Medical Center, and a myeloma partner. The protocol for recruiting and collecting blood samples from allergic subjects was approved by the VUMC Institutional Review Board (IRB 141330 and 142030)[1,2]. ImmunoCAP, ELISA and immunoblotting were used to validate IgE reactivity in donor serum. Further details regarding the allergic donor population and the hIgE mAb panels were published elsewhere[1,2,5]. Herein, four anti-Der p 2 hIgE mAb (1B8, 2G1, 2F10 and 4C8) with distinct epitope specificities were used in pairs to sensitize humanized rat basophilic leukemia cells and to compare the allergenicity of Der p 2 mutants to their WT counterpart.

Passive sensitization protocol

For sensitization with hIgE mAb, individual mAb were combined in equal amounts and diluted in huRBL medium (MEM with Earl's salts without L-glutamine, Sigma-Aldrich (M8042), supplemented with 4 mM L-glutamine, G418 antibiotic and 5% FCSi). For huRBL cell sensitization, the final concentrations per hIgE mAb ranged from 625 to 0.286 IU/mL HDM-specific IgE. To sensitize with allergic donor serum (n = 8), serum was first incubated with Ag-8 cells to inactivate the donor's complement system (to prevent toxic effects on huRBL cells). For this purpose, allergic donor serum was diluted 1:10 in the Ag-8 cell suspension in huRBL cell medium and incubated for 1 h at 37°C. Afterwards, the serum-Ag-8 cell mixture was centrifuged and the supernatant was collected and used to sensitize the huRBL cells, at a final 1:20 dilution of the serum in the 96-well plate. The serum pool consisted of equal parts of the same eight individual sera used in previous assays. HDM allergic donor sera were provided from the CREATE project [6,7], for which all patients provided written consent, and ethical approval was obtained from review board of all participating centers.

Controls for mediator release assays

Controls for the huRBL cell mediator release assay included: wells with non-stimulated, sensitized huRBL cells (sensitized either with hIgE mAb or allergic donor serum), at least two wells containing cells only (background control: unsensitized, unstimulated cells with medium only), and at least two wells for the maximum mediator release control (cells lysed using 1% Triton X-100). Additionally, a control was included where cells were sensitized with an individual hIgE monoclonal antibody and stimulated with WT Der p 2.

Data analysis and statistics

All statistical analyses and data visualizations were performed using Microsoft Excel (version 2016, Microsoft) and GraphPad Prism (version 10.1.2, GraphPad). After background subtraction, results were expressed as a percentage of maximal lysis. To account for assay-to-assay variability due to differences in cell responsiveness and maximal lysis, min-max normalization was applied to each assay, as previously described[8]. HuRBL assays were conducted in technical duplicates or triplicates. Results demonstrating statistical significance were replicated at least three times. Error bars represent the standard deviation of replicated experiments. The limit of quantification (LOQ) for mediator release was determined from the mean of baseline values (background wells) plus 10 times the standard deviation of the baseline and used to define significant mediator release. Area-under-the-curve (AUC) analysis, covering all concentration ranges, was performed by transforming the antigen concentrations (X-axis) of the mediator release data to log(X), with the baseline set to include the lowest transformed value for each dataset. For the AUC from huRBL assay using serum to test the allergenicity of different allergens and inhibition experiments with α-DpX, the baseline was set to 0 and the data between 1 µg/mL and $1x10^{-5}$ µg/mL of the mediator release curves were used. An AUC of 0% mediator release was defined and represented as a reference. Antigen concentrations at 20% mediator release (EC_{20}) were extrapolated using regression line from the mediator release curves. The EC_{20} was used for this comparison, instead of the more commonly used EC_{50}, because the mean of all patients of the double 4C8+2F10 epitope mutant throughout all analysed concentrations did not reach mediator release levels comparable to the EC_{50} observed in the other groups (Figure 3a). Using EC_{20} ensured that all groups could be compared within the dynamic range of the mediator release dose-response curve. Fold difference was calculated using GraphPad Prism by dividing the AUC

produced after stimulation with the mutants by that produced when stimulating with the WT. Significant differences were assessed using either an ordinary one-way/repeated measured one-way ANOVA with multiple comparisons test or an unpaired t test. Statistical significance is indicated by asterisks: $p \le 0.05$ (*), $p \le 0.01$ (**), $p \le 0.001$ (***), and $p \le 0.0001$ (****).

Supplementary Tables

Supplementary Table 1: List of HDM allergic donor sera used to passively sensitized huRBL cells and their levels of HDM sIgE (kU/L).

**CREATE nomenclature**[6,7]	**sIgE (kU/L)**	**Donor serum #**
GOT036	63.2	1
GOT039	25.1	2
GOT040	30.4	3
GOT047	57.8	4
STR017	52.9	5
STR018	24.5	6
STR036	27.4	7
UTR023	33.6	8

Donor serum origin: GOT: Gothenburg, Sweden; STR: Strasbourg, France; UTR: Utrecht, The Netherlands.

Supplementary Table 2: List of Der p 2 specific mAbs used in this study and their concentrations.

**Anti-Der p 2 hIgE mAbs**	
IgE mAb Idiotype	Concentration
2F10	10,000 IU (50,000 IU/mL)
2G1	10,000 IU (50,000 IU/mL)
1B8	10,000 IU (50,000 IU/mL)
4C8	10,000 IU (50,000 IU/mL)
IgG mAb Idiotype	Concentration
αDpX	2 mg/mL

Supplementary Table 3: List of antigens (WT Der p 2 and IgE-epitope mutants).

Species	Antigen and type	Antigen name used in the manuscript
Dermatophagoides pteronyssinus	rDer p 2.0103 wildtype	WT Der p 2
	rDer p 2 4C8 mut AAS	Single 4C8 epitope mutant
	rDer p 2 2F10 mut KKD	Single 2F10 epitope mutant
	rDer p 2 2F10/4C8 mut AAS-KK	Double 4C8+2F10 epitope mutant

Supplementary Table 4: Maximum mediator release (in %) produced by each donor serum after stimulation with either one of the mutants or the WT Der p 2.

Donor serum	WT Der p 2	Single 4C8 epitope mutant	Single 2F10 epitope mutant	Double 2F10+4C8 epitope mutant
#1	100.0	88.1	80.6	77.5
#2	100.0	66.2	99.8	77.2
#3	81.1	100.0	54.7	64.1
#4	100.0	89.3	90.0	36.6
#5	100.0	32.5	27.4	23.7
#6	76.8	70.7	100.0	24.9
#7	100.0	54.4	6.4	2.1
#8	96.3	100.0	61.3	61.0

Supplementary Figures

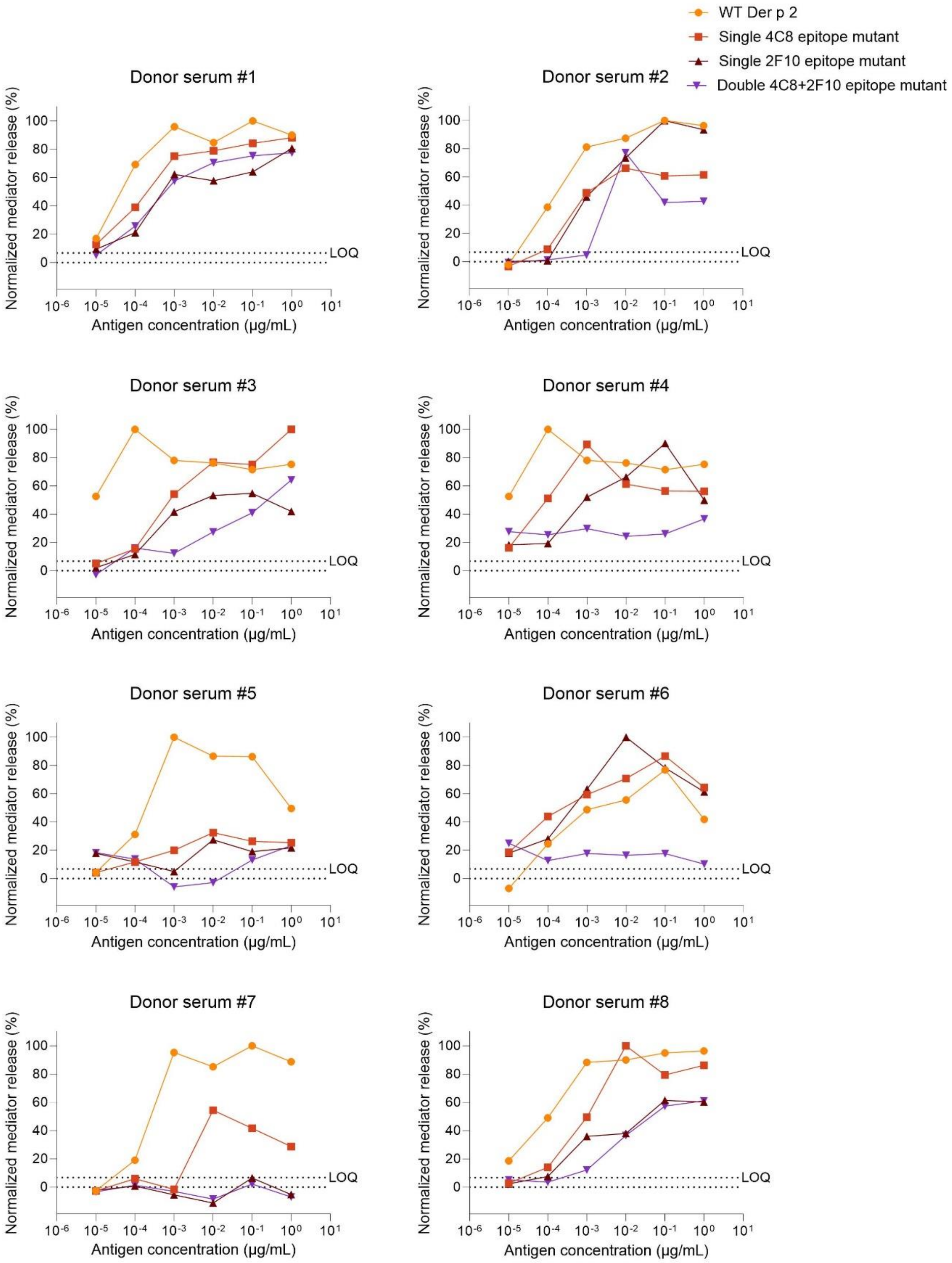

Supplementary Figure 1: Mediator release curves of each donor serum from comparison of Der p 2 IgE-epitope mutants versus WT allergen in stimulating mediator release. HuRBL cells were sensitized with serum from HDM allergic donors **(n=8)** diluted 1:20 in huRBL cell medium and the following day cells were stimulated with a starting concentration of 1 µg/mL of antigen followed by a 1:10 titration. LOQ for the mediator release curve is represented by dotted line as reference.

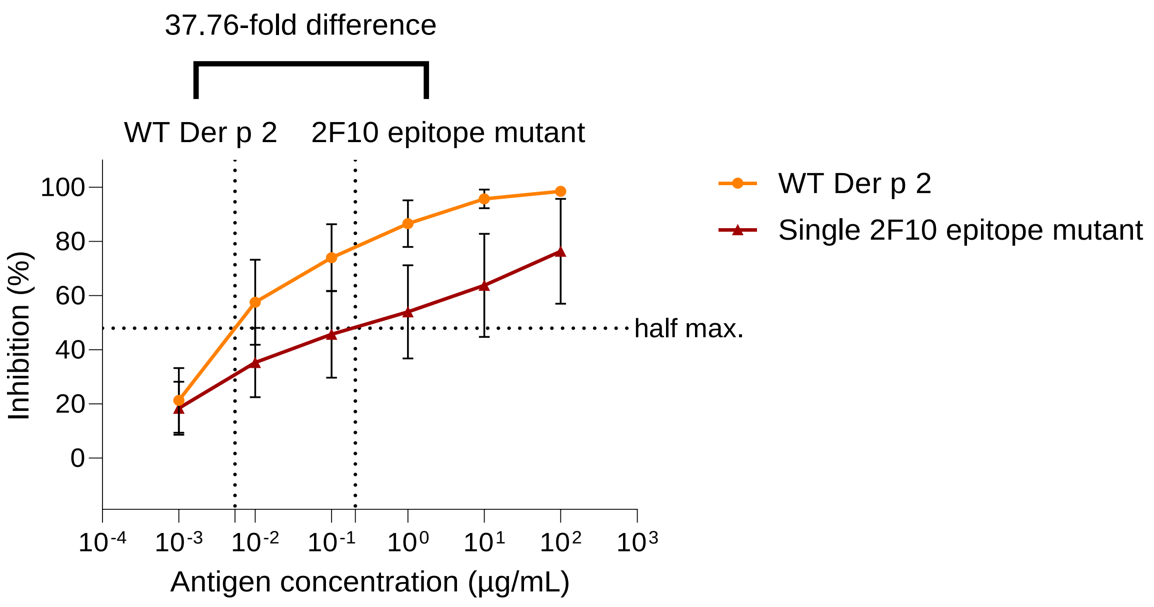

Supplementary Figure 2: Inhibition ELISA using plasma of mite-allergic patients (n=10). Either WT Der p 2 or the single 2F10 epitope mutant were used to inhibit polyclonal IgE antibody binding to Der p 2. The antigen concentration required to achieve half-maximal inhibition (IC_{50}) was determined by interpolating the half-maximal inhibition value onto the logarithmic regression curve. Comparing the antigen concentrations at half-maximal inhibition between WT Der p 2 and the single 2F10 epitope mutant revealed that a 37.76-fold higher antigen concentration is required of the single 2F10 epitope mutant to reach the same level of inhibition as with WT Der p 2. Data were retrieved from Khatri et al.[1], reanalyzed and are shown as mean ± SD of the 10 donors. The half-maximal inhibition value was calculated according to the formula: [half-max. inhibition = ($max_{WT\ Der\ p\ 2}$+$max_{2F10\ mutant}$) ÷ 2]. WT, wildtype

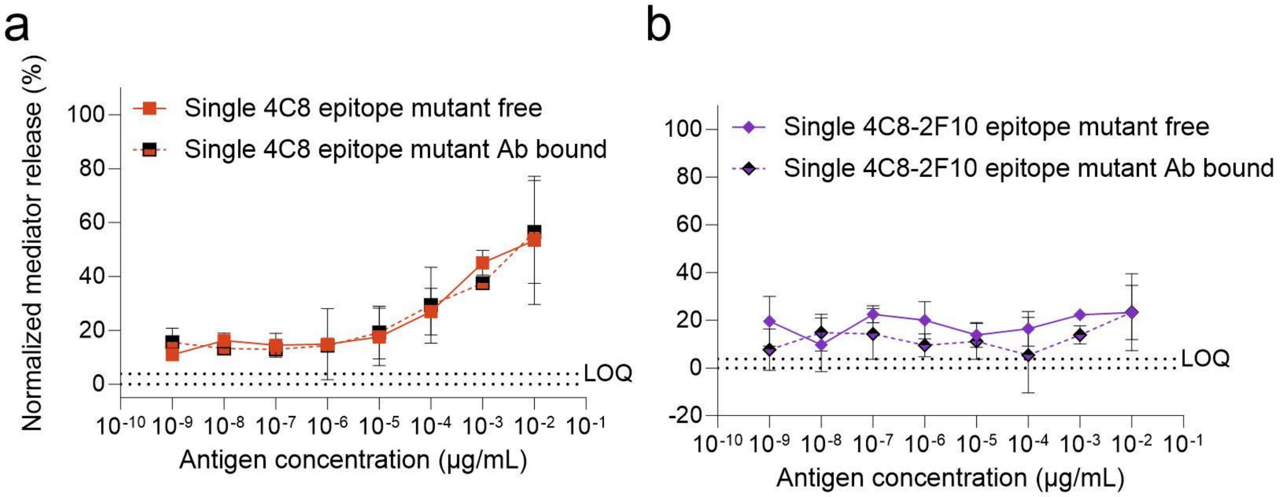

Supplementary Figure 3: Using mIgG mAb αDpX to bind Der p 2 antigens in non-mutated epitope. HuRBL cells were sensitized using a serum pool of previously tested HDM allergic donor serum, diluted 1:20 in huRBL cell medium. The following day, mutants either free or αDpX-bound by mIgG mAb αDpX, were used to stimulate huRBL cells and to induced mediator release. Using mIgG mAb αDpX at a a constant concentration of 2 µg/mL to bind different concentrations of antigen, 1:10 dilution series, starting at 100 ng/mL, representing a 1:1 molar ratio of mIgG mAb-antigen. Normalized mediator release was expressed as percentages (**a-b**). LOQ for the mediator release curve graph is represented by a dotted line as a reference value.

References:

1. Khatri K, Richardson CM, Glesner J, et al. Human IgE monoclonal antibody recognition of mite allergen Der p 2 defines structural basis of an epitope for IgE cross-linking and anaphylaxis in vivo. *PNAS Nexus*. 2022;(June):1-12. doi:10.1093/pnasnexus/pgac054

2. Ball A, Khatri K, Glesner J, et al. Structural analysis of human IgE monoclonal antibody epitopes on dust mite allergen Der p 2. *Journal of Allergy and Clinical Immunology*. 2024;154(2):447-457. doi:10.1016/j.jaci.2024.04.017

3. Wurth MA, Hadadianpour A, Horvath DJ, et al. Human IgE mAbs define variability in commercial Aspergillus extract allergen composition. *JCI Insight*. 2018;3(20). doi:10.1172/jci.insight.123387

4. Mueller GA, Glesner J, Daniel JL, et al. Mapping Human Monoclonal IgE Epitopes on the Major Dust Mite Allergen Der p 2. *The Journal of Immunology*. 2020;205(8):1999-2007. doi:10.4049/jimmunol.2000295cross-linking of the IgE-Fcepsilon receptor comples

5. Smith BRE, Reid Black K, Bermingham M, et al. Unique allergen-specific human IgE monoclonal antibodies derived from patients with allergic disease. *Frontiers in Allergy*. 2023;4. doi:10.3389/falgy.2023.1270326

6. Chapman MD, Ferreira F, Villalba M, et al. The European Union CREATE Project: A model for international standardization of allergy diagnostics and vaccines. *Journal of Allergy and Clinical Immunology*. 2008;122(5). doi:10.1016/j.jaci.2008.07.030

7. Van Ree R, Chapman MD, Ferreira F, et al. The CREATE Project: Development of certified reference materials for allergenic products and validation of methods for their quantification. *Allergy: European Journal of Allergy and Clinical Immunology*. 2008;63(3):310-326. doi:10.1111/j.1398-9995.2007.01612.x

8. Pena-Castellanos G, Smith BRE, Pomés A, et al. Biological activity of human IgE monoclonal antibodies targeting Der p 2, Fel d 1, Ara h 2 in basophil mediator release assays. *Front Immunol*. 2023;14. doi:10.3389/fimmu.2023.1155613
